# Phenobarbital Mediates an Epigenetic Switch at the Constitutive Androstane Receptor (CAR) Target Gene *Cyp2b10* in the Liver of B6C3F1 Mice

**DOI:** 10.1371/journal.pone.0018216

**Published:** 2011-03-24

**Authors:** Harri Lempiäinen, Arne Müller, Sarah Brasa, Soon-Siong Teo, Tim-Christoph Roloff, Laurent Morawiec, Natasa Zamurovic, Axel Vicart, Enrico Funhoff, Philippe Couttet, Dirk Schübeler, Olivier Grenet, Jennifer Marlowe, Jonathan Moggs, Rémi Terranova

**Affiliations:** 1 Investigative Toxicology, Preclinical Safety, Translational Sciences, Novartis Institutes for Biomedical Research, Basel, Switzerland; 2 Friedrich Miescher Institute for Biomedical Research, Basel, Switzerland; University of Illinois at Chicago, United States of America

## Abstract

Evidence suggests that epigenetic perturbations are involved in the adverse effects associated with some drugs and toxicants, including certain classes of non-genotoxic carcinogens. Such epigenetic changes (altered DNA methylation and covalent histone modifications) may take place at the earliest stages of carcinogenesis and their identification holds great promise for biomedical research. Here, we evaluate the sensitivity and specificity of genome-wide epigenomic and transcriptomic profiling in phenobarbital (PB)-treated B6C3F1 mice, a well-characterized rodent model of non-genotoxic liver carcinogenesis. Methylated DNA Immunoprecipitation (MeDIP)-coupled microarray profiling of 17,967 promoter regions and 4,566 intergenic CpG islands was combined with genome-wide mRNA expression profiling to identify liver tissue-specific PB-mediated DNA methylation and transcriptional alterations. Only a limited number of significant anti-correlations were observed between PB-induced transcriptional and promoter-based DNA methylation perturbations. However, the constitutive androstane receptor (CAR) target gene *Cyp2b10* was found to be concomitantly hypomethylated and transcriptionally activated in a liver tissue-specific manner following PB treatment. Furthermore, analysis of active and repressive histone modifications using chromatin immunoprecipitation revealed a strong PB-mediated epigenetic switch at the *Cyp2b10* promoter. Our data reveal that PB-induced transcriptional perturbations are not generally associated with broad changes in the DNA methylation status at proximal promoters and suggest that the drug-inducible CAR pathway regulates an epigenetic switch from repressive to active chromatin at the target gene *Cyp2b10*. This study demonstrates the utility of integrated epigenomic and transcriptomic profiling for elucidating early mechanisms and biomarkers of non-genotoxic carcinogenesis.

## Introduction

The coordinated interplay of different layers of epigenetic regulation (such as DNA methylation and post-translational modifications (PTMs) of histone proteins) provide a dynamic platform for functionally organizing the genome in a developmental-stage and cell-type specific manner [1], [2], [3], [4], [5], [6], [7], [8], [9]. The term “epigenetics” describes changes in gene activity in the absence of a change in DNA sequence and was recently defined as *“the structural adaptation of chromosomal regions so as to register, signal or perpetuate altered activity states”* [10]. This definition does not take into account whether epigenetic modifications are heritable or causal. Recent research has begun to unravel the molecular basis for how cells read and write epigenetic codes and has also revealed a close association between epigenetic changes and the predisposition to, and development of, a wide range of human diseases [11].

The epigenetic landscape of cancer cells is highly distorted. Global reduction in DNA methylation and global alterations in histone PTMs have been identified as general features of neoplasia [12], [13], [14], [15], [16]. However, the key molecular events leading to carcinogenesis remain poorly characterized. Chromatin alterations at individual gene promoters, including many growth-promoting and tumor-suppressor genes, at the earliest stages of tumor development and prior to detectable chromosomal alterations are associated with aberrant gene regulation. For example, promoter hypermethylation has been detected in non-progressed adenomas in which no chromosomal alterations exist (Derks et al. 2006), suggesting that early epigenetic events contribute to gene expression changes during tumor progression. Aberrant CpG island methylation also tends to accumulate during the course of multistage carcinogenesis (Kang et al. 2003). Early epigenetic aberrations have been proposed to contribute to the transformed phenotype by promoting the expansion of pre-malignant cells during the earliest stages of tumorigenesis [17], [18]. Further evidence, including the reversibility of the tumor phenotype following experimental reprogramming, support a role for epigenetic alterations in cancer [18]. Together, these observations have resulted in a paradigm shift in our understanding of mechanisms of carcinogenesis involving both epigenetic plasticity and genetic lesions at each stage (initiation, promotion and progression) of carcinogenesis [18], [19].

Epigenetic perturbations may also be involved in the adverse effects associated with some drugs and toxicants, including certain classes of non-genotoxic carcinogens [20], [21], [22], [23]. For example, drug-induced stress (e.g. chronic injury/inflammation/reactive oxygen species) may trigger epigenetic changes that “lock-in” abnormal proliferative states via heritable transcriptional repression of key genes/pathways [18]. Thus, epigenomic profiling has great potential for enhancing our understanding of the molecular basis of spontaneous or drug-mediated aberrant cell cycle and apoptosis regulation in cancer. A wide range of novel epigenomic profiling technologies for both DNA methylation and histone modification analysis have been developed in recent years [24], and application of these technologies provides a unique opportunity for mechanistic insights and biomarker identification during both preclinical and clinical phases of drug development [21], [25].

Phenobarbital (PB), the most widely used anticonvulsant worldwide, is a well established rodent non-genotoxic carcinogen that functions as a tumor promoter, increasing the incidence of spontaneously and chemically induced tumors in a strain-specific manner [26], [27], [28], [29]. PB accomplishes its diverse effects on liver function in part by promoting a nuclear translocation of the constitutive androstane receptor (CAR) [30]. The CAR receptor can be activated by numerous therapeutics, constituting a central defense mechanism against their toxicity and carcinogenicity [31]. CAR is required for gene expression changes, hepatomegaly and liver tumor formation elicited by prolonged PB treatment in mice [32], [33]. Prolonged PB treatment (0.05% w/v in drinking water for 12 months) significantly promotes hepatic tumor incidence in B6C3F1 mice (from 29% in the absence of PB to 100% following PB promotion), as well as increases the size and number of tumors in every treated animal [29], possibly via the growth promotion of spontaneously initiated hepatocytes (for review, see [28]). This PB-mediated tumor promotion model provides an excellent system in which epigenetic and transcriptional alterations can be profiled at different stages during promotion and progression of the carcinogenic process. A number of studies have identified progressive, non-random changes in DNA methylation and gene expression both at early stages (2–4 weeks, in the absence of mutagenic initiation) and later stages (23–32 weeks following diethylnitrosamine (DEN) initiation) of PB treatment in B6C3F1 mice [32], [34], [35], [36]. It has yet to be determined whether these alterations are causal, associative, or incidental to carcinogenesis. In this study we evaluate the general utility of genome-wide and locus-specific DNA methylation assays in a mouse model with particular emphasis on the identification of early mechanism-based markers for non-genotoxic carcinogenesis (NGC). We use genome-wide DNA methylation and transcriptome profiling of liver (target) and kidney (non-target) tissues of B6C3F1 mice after 4 weeks of PB treatment, in the absence of chemical initiation (as in [35]). We identify PB-mediated tissue-specific transcriptional and promoter DNA methylation changes. We show that *Cyp2b10*, a direct and early target of the CAR pathway [37], is concomitantly hypomethylated and activated in a tissue-specific manner following PB treatment, and further characterize an epigenetic switch from a repressive to an active chromatin configuration at this locus. The application and extension of epigenomic profiling approaches will contribute to a better understanding of mechanisms of non-genotoxic carcinogenesis and may lead to the identification of powerful predictive biomarkers of NGC.

## Results

### 4-week PB treatment leads to tissue-specific transcriptional remodeling

**Figure 1:**
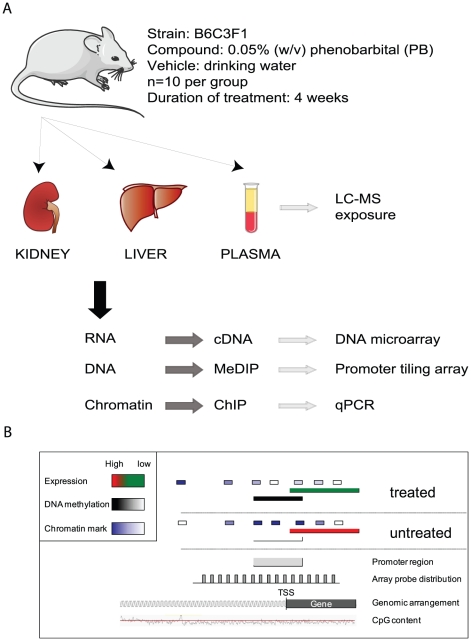
Overview of study design and data integration. (**A**) Experimental strategy for the identification and integration of PB-induced expression and epigenetic perturbations in target (liver) and non-target (kidney) tissues. RNA, DNA and chromatin was extracted from liver and kidney samples of control and PB-treated (4-week, 0.05% in drinking water) B6C3F1 male mice (n = 10 per group) and analyzed through the different profiling methodologies and platforms indicated. Abbreviations: liquid chromatography-mass spectrometry (LC-MS), Methylated DNA immunoprecipitation (MeDIP), Chromatin immunoprecipitation (ChIP), quantitative real-time PCR (qPCR). (**B**) Summary of bioinformatic data integration strategy. For each annotated gene present on gene expression and promoter arrays, the expression and DNA methylation values were mapped to the genome and correlated to examine the functional links between expression and methylation levels at individual loci upon phenobarbital treatment. For loci of interest the abundance of selected chromatin marks were quantified. Coverage of the promoter array (1.8 kb per promoter: 500 bp downstream and 1300 bp upstream of the transcriptional start site (TSS)) is shown. For the methylation analysis a window of 100 bp downstream and 800 bp upstream of the TSS was used. The figure shows an exemplary gene that upon treatment loses DNA methylation and gets expressed.

Prolonged PB treatment (0.05% w/v in drinking water for 12 months) results in 100% hepatic tumor incidence in B6C3F1 mice [29]. To gain insights into the early events leading to liver carcinogenesis and to identify early and potentially predictive promoter-specific epigenetic changes associated with the early stages of carcinogenesis, we treated B6C3F1 mice in conditions reported to lead to transcriptional and epigenetic perturbations [34], [35]. Mice were given *ad libitum* access to drinking water containing 0.05% (w/v) phenobarbital for a period of 28 days. Total RNA from liver and kidney was purified and processed for Affymetrix gene expression profiling while genomic DNA was prepared for promoter array based methylome analysis using the Methylated DNA immunoprecipitation (MeDIP) procedure [38]. Remaining tissue material was used for chromatin immunoprecipitation (ChIP) to analyze histone modifications at individual promoters. Plasma samples were also collected to evaluate phenobarbital exposure in individual animals by LC-MS, and showed consistent systemic drug exposure for all 10 animals used in this study (data not shown). An overview of the experimental system and bioinformatics pipeline integrating transcriptomic and epigenetic data is summarized in Figures 1A and 1B.

Affymetrix microarray datasets were generated from the liver and kidney of all individual control and treated animals. Transcription profiling of the liver identified 349 probes, representing 231 genes, with significantly altered expression after 4-week PB treatment (see methods for details). MAS5 normalized microarray data is provided as Supplementary Tables S1, S2, S4 and S6, and the top 30 differentially up and down regulated genes are shown in Table 1 and 2. Hierarchical clustering grouped these 349 probes into coregulated clusters (Figure S1A). The clustering perfectly separated the control and the PB-treated samples into two respective groups, supporting reproducibility in expression changes. Overall more genes were upregulated (150) than downregulated (81) upon PB treatment. To gain insight into the predominant pathways and functions altered by PB-treatment, we interrogated the 231 genes using Ingenuity Pathway Analysis (version 8.6; Ingenuity Systems; www.ingenuity.com). Differentially regulated genes were strongly enriched in functional categories associated with small molecule biochemistry, lipid metabolism, and drug metabolism, including the metabolism of xenobiotics by cytochromes P450 (Figure S1B, Table S3). 20 out of the top-30 upregulated genes (Table 1) were previously listed as PB-inducible using identical PB-treatment conditions in B6C3F1 [35]. In addition to the classical PB-induced genes (like *Cyp2b10, Gsta2, Gstt3*) several other genes show consistent upregulation in both data sets (including *Orm3, Akr1b7, Lect1, A930034L06Rik, Gadd45b, Prom1, A930034L06Rik, Meig1, Pnliprp1, Wisp1 and Cxcr7*). Many of these upregulated genes, like *Gadd45b* and *Wisp1*, influence cell cycle/death and are associated with cancer. It is noteworthy that we detected a significant five-fold upregulation of *Cyp2b9*, a gene previously reported as non-PB-inducible gene in mouse liver [39] and used as a negative control in the studies that led to the discovery of the role of CAR in *Cyp2b10* regulation [37], [40]. Differences in experimental conditions and/or transcript detection methodologies might account for this discrepancy. We also identified, to our knowledge, novel PB-induced transcriptional perturbations including significant up-regulation of the *Nebl* (*Nebulette*) gene that codes for a component of cardiac muscle fibers [41] and whose liver-specific functions remain to be determined. In addition, only 3 out of the 30 downregulated genes (namely *Dnaic1, Slc41a2* and *Csad*, Table 2) were previously reported to be perturbed using identical PB-treatment conditions in B6C3F1 [35]. These minor discrepancies in PB-induced transcriptional perturbations within the same strain of mice are likely to be due to a number of factors including *in vivo* study conditions, laboratory-specific sample handling and data processing methods (as reported in Leek et al. [42]).Transcriptional profiling of kidney samples identified only 53 significantly altered probe sets, representing 39 genes (Table S4), none of which were significantly changed in liver (p = 0.001), highlighting the tissue-specificity of PB-induced changes.

**Table 1:** Correlating gene expression and DNA methylation PB-induced perturbations in the liver of B6C3F1 mice.

Gene Symbol	Gene Expression		DNA Methylation		Correlation		CpG class
	Fold change	p value	Log2 ΔMeth	p value	Corr	p value	
Cyp2b10	451.86	8.20E-11	-0.305	1.17E-08	-0.931	2.56E-09	CpG poor
Cyp2c55	32.42	2.18E-10	-0.016	5.42E-01	-0.163	4.94E-01	CpG poor
Nebl	31.01	5.66E-08	-0.040	1.57E-01	-0.227	3.37E-01	strong CpG island
Meig1	10.44	1.04E-06	0.010	8.18E-01	0.138	5.63E-01	weak CpG island
Pnliprp1	10.05	1.11E-07	-0.061	1.94E-02	-0.460	4.13E-02	CpG poor
Akr1b7	9.37	4.15E-07	0.026	3.74E-01	0.221	3.49E-01	CpG poor
Sult1e1	9.09	2.52E-05	0.029	4.91E-01	0.227	3.35E-01	weak CpG island
Serpinb1a	8.86	2.34E-05	0.005	9.07E-01	0.087	7.14E-01	CpG poor
Gstm3	8.76	3.55E-05	-0.032	3.68E-01	-0.195	4.11E-01	CpG poor
Gstt3	7.63	2.12E-08	0.009	8.17E-01	0.002	9.92E-01	weak CpG island
Lect1	7.59	4.29E-09	-0.003	9.09E-01	-0.053	8.23E-01	weak CpG island
Cyp2d12	7.30	1.73E-05	0.013	6.91E-01	0.214	3.65E-01	CpG poor
AW125753	7.18	3.37E-02	-0.051	6.81E-02	-0.426	6.13E-02	strong CpG island
Orm3	6.79	9.63E-06	-0.069	1.17E-01	-0.444	4.96E-02	CpG poor
Gadd45b	6.77	1.39E-05	0.028	4.42E-01	-0.016	9.48E-01	strong CpG island
Cyp2b9	5.69	3.20E-04	-0.035	3.01E-01	-0.214	3.64E-01	CpG poor
9630050M13Rik	5.65	8.92E-07	-0.035	4.61E-01	-0.099	6.78E-01	strong CpG island
Cyp2c37	5.48	5.07E-14	0.057	2.01E-01	0.262	2.64E-01	CpG poor
Dbp	5.31	2.57E-02	0.017	6.76E-01	0.521	1.86E-02	weak CpG island
Cyp2c54	5.13	1.98E-10	0.102	2.11E-02	0.472	3.55E-02	CpG poor
1700067K01Rik	4.98	2.48E-11	-0.026	2.73E-01	-0.233	3.22E-01	strong CpG island
Wisp1	4.75	9.03E-07	-0.004	8.88E-01	-0.171	4.70E-01	CpG poor
Gsta1	4.65	7.36E-05	0.030	2.80E-01	0.052	8.28E-01	CpG poor
Prom1	4.51	4.72E-04	0.005	8.74E-01	0.132	5.79E-01	weak CpG island
Kcnk1	4.39	5.88E-04	0.035	8.08E-02	0.283	2.26E-01	strong CpG island
Gstm1	4.30	2.07E-08	-0.041	2.75E-01	-0.329	1.57E-01	weak CpG island
Gsta2	4.29	5.99E-06	0.015	6.34E-01	0.034	8.87E-01	CpG poor
Cxcr7	4.29	2.72E-04	0.033	1.91E-01	0.461	4.10E-02	weak CpG island
A930034L06Rik	3.35	2.31E-08	0.035	3.05E-01	0.110	6.44E-01	weak CpG island
Por	3.35	1.59E-08	0.050	7.59E-02	0.306	1.90E-01	strong CpG island

Overview of the Top-30 most differentially up-regulated genes (for genes with MAS5 expression value>50). The linear expression fold change and delta methylation (Log2 ΔMeth) are correlated. For the genes with several Affymetrix probesets showing strong transcriptional change only the value for the probe set with highest MAS5 value in the control samples is shown in this table. The full list of genes (30,430 Affymetrix probe Sets) is available in Table S6.

**Table 2:** Overview of the Top-30 most differentially down-regulated genes (for genes with MAS5 expression value>50).

Gene Symbol	Gene Expression		DNA Methylation		Correlation		CpG class
	Fold change	p value	Log2 ΔMeth	p value	Corr	p value	
Mt2	0.09	2.23E-02	0.065	1.99E-01	0.060	8.03E-01	strong CpG island
Egr1	0.14	2.68E-04	-0.028	1.49E-01	0.381	9.77E-02	strong CpG island
Mt1	0.18	3.41E-02	0.057	5.25E-02	-0.407	7.50E-02	strong CpG island
Fabp5	0.18	2.06E-06	0.003	9.27E-01	-0.070	7.69E-01	weak CpG island
Cdkn1a	0.19	2.16E-03	-0.003	8.88E-01	0.101	6.71E-01	strong CpG island
Igfbp1	0.21	9.62E-03	0.000	9.88E-01	-0.263	2.64E-01	weak CpG island
Fos	0.22	3.38E-02	0.033	8.45E-02	-0.326	1.60E-01	strong CpG island
Cxcl1	0.24	2.94E-02	0.077	4.78E-02	-0.148	5.33E-01	weak CpG island
Kap	0.25	3.75E-01	0.029	4.44E-01	0.031	8.96E-01	CpG poor
Scara5	0.27	6.33E-04	-0.016	6.31E-01	-0.016	9.45E-01	CpG poor
1600029D21Rik	0.27	2.00E-01	0.057	2.51E-01	0.305	1.91E-01	weak CpG island
Gnat1	0.28	1.61E-08	0.000	9.89E-01	-0.075	7.54E-01	CpG poor
Tnfrsf12a	0.29	1.19E-01	0.011	7.03E-01	-0.329	1.57E-01	strong CpG island
3110082I17Rik	0.31	3.21E-03	0.012	7.56E-01	-0.003	9.91E-01	weak CpG island
Dnaic1	0.32	3.06E-05	-0.021	5.31E-01	0.134	5.72E-01	strong CpG island
Il33	0.32	2.58E-01	0.110	3.71E-02	-0.251	2.85E-01	weak CpG island
Steap4	0.32	1.07E-03	0.004	8.33E-01	0.072	7.64E-01	weak CpG island
Il1r1	0.32	2.93E-03	-0.047	2.62E-01	0.264	2.62E-01	weak CpG island
Socs2	0.33	2.22E-02	0.016	5.98E-01	-0.102	6.68E-01	strong CpG island
2010003K11Rik	0.33	1.36E-01	-0.023	4.80E-01	-0.165	4.88E-01	CpG poor
Lpin1	0.34	3.23E-03	0.028	2.85E-01	-0.221	3.49E-01	weak CpG island
Saa4	0.34	2.96E-03	0.060	4.60E-02	-0.376	1.02E-01	CpG poor
B3galt1	0.36	1.95E-03	-0.004	9.42E-01	0.056	8.13E-01	CpG poor
Slc41a2	0.36	2.07E-05	-0.029	3.73E-01	-0.021	9.29E-01	strong CpG island
Csad	0.39	1.12E-06	0.048	3.01E-01	-0.065	7.85E-01	CpG poor
Egfr	0.40	7.73E-04	0.036	2.30E-01	-0.108	6.49E-01	strong CpG island
Plscr1	0.40	2.29E-01	0.022	6.53E-01	0.382	9.67E-02	CpG poor
Clpx	0.40	5.83E-03	0.034	6.59E-02	-0.118	6.21E-01	strong CpG island
Foxq1	0.41	5.18E-02	0.022	4.13E-01	-0.397	8.34E-02	strong CpG island
Gadd45g	0.42	7.70E-02	-0.052	3.12E-02	0.298	2.02E-01	strong CpG island

As in Table1.

### Genome-wide MeDIP profiling identifies tissue-specific PB-induced DNA methylation perturbations

**Figure 2:**
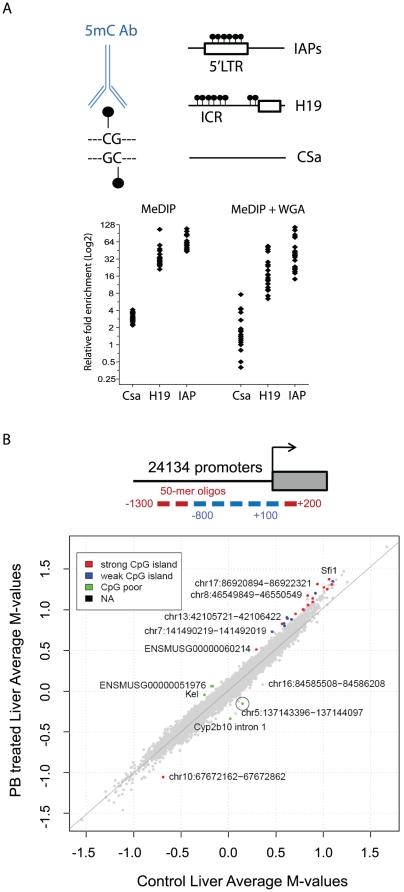
MeDIP-promoter array profiling of the methylome in the liver of control and PB treated B6C3F1 mice. (**A**) An antibody directed against 5-methyl-cytosine (5mC) was used for immunoprecipitation of methylated DNA. Control sequences that are highly methylated (IAP, H19 ICR) or lack CpGs (CSa) were selected as controls for the MeDIP experiment prior to and following whole genome amplification (WGA). The relative enrichment in the bound over input fractions for 10 individual biological replicates was measured by qPCR. (**B**) Methylation comparison between liver of control and PB-treated mice (average log_2_ (IP/total) for replicates) in all 23,428 Nimblegen probe sets. The colors indicate the CpG island class for those probe sets where the log_2_ methylation ratio of PB-treated vs. control (difference in M-value) is significant (p≤0.01 and an absolute log_2_ fold change of ≥0.2, 28 probe sets), non-differentially methylated regions are indicated in grey. A circle highlights *Cyp2b10*.

Epigenetic changes may precede or underlie PB-induced transcriptional remodeling in the liver, providing mechanistic insights as well as a rich source of biomarkers of the carcinogenic process. MeDIP was combined with large-scale promoter tiling-array to examine genome-wide PB-induced DNA methylation perturbations. Methylated DNA from liver (target) and kidney (non-target) was isolated from all PB-treated and control animals. To control for the validity of the methylated DNA enrichment procedure, regions known to be enriched in DNA methylation (such as the *H19* imprinting control region (ICR) or the intracisternal A-particle element (*IAP*)), or devoid of methylation (*CSa*) were profiled by real-time PCR (qPCR) prior to and following whole-genome amplification (WGA). This control indicates consistent enrichment of methylated genomic regions in all samples prior to and following WGA amplification (Figure 2A).

MeDIP-enriched and input genomic DNA was labeled with different fluorescent dyes and hybridized to a Nimblegen promoter array covering 1.8 kb (1.3 kb upstream and 0.5 kb downstream) of the transcription start site (TSS) of 17,967 genes and additional 4,566 intergenic CpG islands (385K CpG Island Plus Promoter Array, Nimblegen, see methods). Array datasets were generated in duplicate for liver and kidney of all control and treated animals. The ratio of methylated to input signal was calculated for each sequence spotted on the array, values for all probes in each promoter were summarized and used as a read-out for the methylation level as previously reported [3].

Statistical analysis (ANOVA p≤0.001, see Processing Nimblegen chips in the methods section) identified 12,362 probe sets with significant differential methylation between the organs, 163 with an overall treatment effect (across organs) and 2,520 with organ and treatment interactions, indicating that the liver and kidney methylomes are very different (see also Figure S2), and the effect of PB-treatment is organ specific.

**Figure 3:**
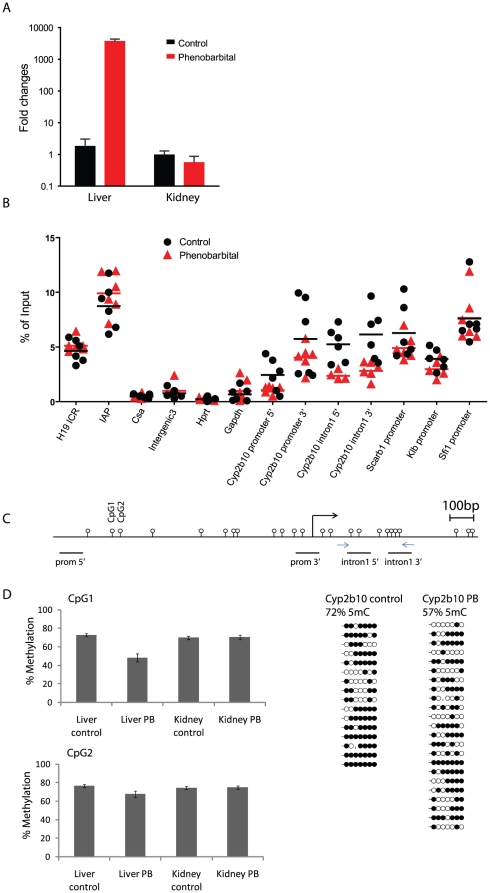
*Cyp2b10* is selectively DNA demethylated and over expressed in the liver of PB-treated B6C3F1 mice. (**A**) RT-qPCR analysis of Cy2b10 expression in the liver and the kidney of control and PB-treated animals. Fold changes are indicated relative to 18S RNA expression levels. (**B**) MeDIP-qPCR validation of Nimblegen data. Positive (H19 ICR, IAP), negative (CSa, Intergenic3, Hprt, Gapdh) and selected regions identified on the promoter array were assessed by qPCR from WGA amplified MeDIP and input DNA samples prepared from the liver of 6 control and PB-treated mice. qPCR identified selective demethylation at *Cyp2b10* TSS, both in the promoter and first intron (location of PCR amplicons is shown in Figure 3C). Relative enrichment (IP/Input) for DNA methylation of 6 individual biological replicates is shown. (**C**) Bisulfite sequencing at *Cyp2b10* first intron. Sequenced area is shown by the two arrows in the schematic gene map. Each line represents the sequence of a single clone. CpGs are shown as white (unmethylated) or black (methylated) circles. The values above summarizes the overall methylation level of this region (percentage of methylated CpG in all sequenced clones) (**D**) Quantitative DNA methylation analysis by pyrosequencing of two *Cyp2b10* promoter CpG sites (CpG1: -914 and CpG2: -886 indicated in (C)) in the liver and kidney of control and PB-treated animals. Standard deviation was calculated from 10 biological replicates. Primers/genomic regions used for bisulfite sequencing are available in Figure S9.

In liver, we identified 28 probe sets with statistically significant (p≤0.01 and absolute log_2_ fold change ≥0.2) changes in DNA methylation (Figure S2A and Table S5). The integration of transcriptional and promoter-methylation data failed to identify a significant overall anti-correlation between gene expression and methylation alterations in liver (Table 1 and 2 and Table S6), with the exception of *Cyp2b10*, a well-characterized CAR target gene, which was identified as the most up regulated gene and most significantly demethylated promoter, specifically in liver tissue (Table 1, Table S6 and Figure S3A). This functional anti-correlation was further validated through qPCR analysis of *Cyp2b10* expression level in liver and kidney, showing a robust liver-specific increase in *Cyp2b10* expression level (Figure 3A). Methylome profiling in kidney identified 286 differentially methylated regions (with the same set of filters as for liver) (Figures S2A, S2B), including 5 hypomethylated probe sets which are hypermethylated in liver (Sfi1 and 4 unannotated probe sets). In comparison, 2811 probe sets are differentially methylated when comparing the methylomes of control kidney and liver (Figure S2C). Thus, PB treatment leads to a relatively limited number of promoter DNA methylation perturbations, which show high tissue specificity. Our results suggest that most changes in gene expression are not associated with promoter-wide methylation alterations upon phenobarbital treatment. We cannot however exclude that DNA methylation changes at single or selected CpGs within promoters may lead to transcriptional perturbations as the MeDIP-array approach is likely not sensitive enough to detect very discrete alterations.

### PB-treatment leads to *Cyp2b10* promoter demethylation in the liver of B6C3F1 mice

To independently confirm observed promoter changes in DNA methylation, qPCR assays were designed for selected genes. Control sequences that are highly methylated (*H19 ICR, IAP*), unmethylated (*Intergenic region3, Hprt, Gapdh promoters*) or lack CpGs (*CSa*) were used as controls for the methylation status of specific promoter regions in control and PB-treated animals (n = 6). The qPCR analysis confirmed *Cyp2b10* demethylation in PB-treated animals. In contrast, no consistent change in DNA methylation was detected by qPCR at the promoters of *Sfi1*, *Scarb1* and *Klotho-β*(*Klb*), three regions showing relatively high PB-induced DNA methylation changes on the promoter-arrays (Tables S5 and S6).

The promoter array covers 1.8 kb per TSS while the bioinformatic analyses focused on a 900 bp window (100 bp downstream and 800 bp upstream of the TSS). Further investigation of the *Cyp2b10* locus identified the first intron of *Cyp2b10* as the region showing the most significant DNA demethylation (Figure S3B), an observation validated through qPCR on MeDIP-enriched DNA (Figure 3B).

To further characterize DNA demethylation at the *Cyp2b10* TSS, genomic DNA from control and PB-treated animals was bisulfite converted and the methylation status of individual CpGs analyzed. A region containing 7 CpGs in the first intron of *Cyp2b10* was amplified and cloned. The sequencing of individual clones showed variable levels of DNA methylation at different CpG sites. The overall methylation level of this region was calculated and showed a 15% decrease in DNA methylation (72% and 57% methylated CpGs in control and PB treated samples, respectively) (Figure 3C). Two cytosines in the *Cyp2b10* promoter region (-886 and -916 from *Cyp2b10* transcription start site) were further selected for quantitative pyrosequencing assessment of methylation level in liver and kidney upon PB treatment. This experiment identified a PB-mediated 25% and 9% reduction in DNA methylation of CpG1 (-916) and CpG2 (-886), respectively, specifically in the liver (Figure 3D). These results altogether support tissue-specific, PB-mediated DNA methylation perturbation of the *Cyp2b10* promoter.

### Native Chromatin immunoprecipitation identifies an epigenetic switch at *Cyp2b10* TSS

To assess the extent of global epigenetic/protein remodeling in the liver and kidney upon PB treatment, a reverse protein array approach (RPA) was used. This enabled us to profile the relative levels of 16 histone PTMs and 31 (phospho)-proteins (Figure S4 and Tables S7 and S8). No significant difference in global abundance was observed in livers of 8 control and 8 PB-treated animals for a range of chromatin marks (acetylation and methylation) associated with activation and repression, as well as in a range of proteins involved in different regulatory pathways.

**Figure 4:**
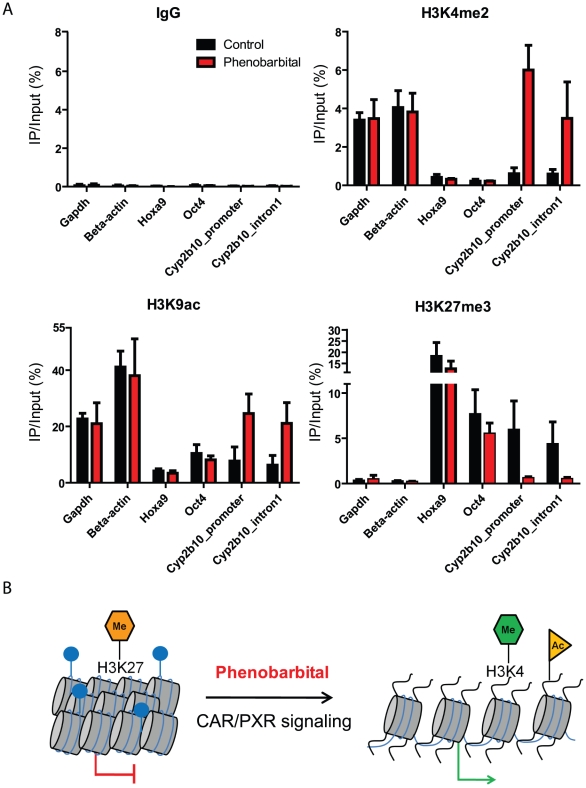
Native chromatin immunoprecipitation identifies a PB-induced epigenetic switch at *Cyp2b10* transcriptional start site (TSS). (**A**) Following nuclei purification and micrococcal nuclease treatment, chromatin was immunoprecipitated with antibodies directed against the active marks H3K4me2 and H3K9ac or the repressive mark H3K27me3. IgG was used as a negative IP control in these experiments. The relative enrichment for different marks was analyzed around *Cyp2b10* TSS. Ubiquitously expressed, active (Gapdh, beta-actin) and pluripotency associated, repressed (Hoxa9, Oct4) genes were used to control the selectivity of antibodies. Standard deviation was calculated from 3 (H3K9ac) and 5 (IgG, H3K4me2 and H3K27me3) biological replicates. (**B**) Model representing the epigenetic switch at the *Cyp2b10* TSS upon 4 weeks of PB treatment. Blue circles represent methylated cytosines.

To ask if changes occurred locally without effecting global abundance, we performed native chromatin immunoprecipitation (N-ChIP) on selected liver samples. Nuclei from individual liver samples were prepared, the chromatin was fractionated using micrococcal nuclease [43] and samples were enriched for fractions associated with transcriptional activity (acetylation of lysine 9 of histone H3 (H3K9ac) or dimethylation of lysine 4 of histone H3 (H3K4me2)) and transcriptional repression (trimethylation of lysine 27 of histone H3 (H3K27me3)) using histone-modification specific antibodies. This experiment identified a strong epigenetic switch at the *Cyp2b10* TSS region, from a repressive (H3K27me3 rich) to an active (H3K4me2, H3K9ac rich, H3K27me3 poor) chromatin configuration following 4 weeks of PB treatment (Figures 4A and 4B). Constitutively activated genes (*Gapdh, beta-actin*) and pluripotency associated (repressed) genes (*Hoxa9, Oct4*) showed no chromatin changes in this analysis and served as controls (Figure 4A).

## Discussion

The working hypothesis for the mechanistic investigation outlined here is that epigenetic modifications, namely DNA methylation, will provide valuable insights into early molecular mechanisms and reveal potential biomarkers of non-genotoxic carcinogenesis (NGC). In this study we evaluate the general utility of genome-wide and locus-specific DNA methylation assays in preclinical animal models with particular emphasis on the identification of early mechanism-based markers of NGC in rodents. This study examines genome-wide tissue-specific gene expression and promoter/CpG island-specific DNA methylation changes upon short term exposure to a well-characterized rodent non-genotoxic hepatocarcinogen. We use a well-characterized *in vivo* 4-week PB-treatment protocol [35] that has been previously used to investigate early epigenetic mechanisms associated with NGC. We have identified liver tissue-specific PB-mediated DNA methylation and transcriptional alterations using MeDIP-coupled microarray profiling of DNA methylation combined with genome-wide mRNA expression profiling. Our manuscript describes several novel aspects of PB-induced molecular responses in the mouse. First, we present an integrated genome-wide view of PB-induced perturbations of mRNA transcription and promoter DNA methylation in which the DNA methylation status of all known mouse promoters and CpG islands can be interrogated in a locus-specific manner. Second, we describe for the first time, robust liver-specific PB-induced DNA methylation changes within the promoter and first intron of *Cyp2b10*. Furthermore, we provide for the first time an integrated view of PB-induced alterations in *Cyp2b10* mRNA transcription, DNA methylation and histone post-translational modifications (both active and silent marks). This integrated molecular profiling approach can in principle be applied to any loci of interest and provides a powerful method for elucidating early mechanisms and pathways underlying non-genotoxic carcinogenesis.

Our genome-wide data show a limited number of significant anti-correlations between transcriptional changes and promoter-based DNA methylation perturbations in both liver and kidney. It is reported that epigenetic perturbations may precede, parallel or follow transcriptional perturbations and can be uncoupled from alterations in transcriptional activity [44]. Such epigenetic changes that are uncoupled in time from transcriptional alterations could be potential biomarkers and contribute to lasting physiological changes that only later manifest in transcriptional changes. In this respect the limited correlation between promoter DNA methylation and transcriptional perturbations observed in this study is an important concept for the identification of early NGC epigenetic biomarkers. PB is a liver (and not a kidney) non-genotoxic carcinogen [26], [45], [46]. Whilst it is somewhat surprising that more significant DNA methylation changes are detected in the kidney than in liver, the lack of overlapping PB-induced changes in DNA methylation changes between liver and kidney tissues is consistent with a previous B6C3F1 mouse PB 4-week study in which the methylation status of GC-rich regions of DNA was assessed via methylation-sensitive restriction digestion, arbitrarily primed polymerase chain reaction, and capillary electrophoretic separation of products [34]. These authors reported that PB-induced alterations in liver DNA methylation were highly dissimilar to those of kidney [34]. Collectively these observations may help better differentiate between early DNA methylation perturbations that may be associated with carcinogenic versus non-carcinogenic molecular pathways.

We found *Cyp2b10*, a known CAR target, to be concomitantly hypomethylated and activated in a tissue-specific manner following PB treatment and revealed a PB-mediated epigenetic switch at the promoter of this gene. Whether the DNA methylation and histone modification changes observed at *Cyp2b10* TSS are a cause or consequence of the transcriptional induction of *Cyp2b10* remains to be determined. Further *in vivo* mechanistic/profiling analyses at very early time points following PB exposure, prior to *Cyp2b10* transcriptional activation will be required to decipher the sequence of events and epigenetic mechanisms associated with *Cyp2b10* activation. Previous studies have shown that 4 hours of PB treatment in mice can trigger *Cyp2b10* upregulation [47], suggesting that a rapid epigenetic switch may take place at this promoter following PB exposure and CAR activation. The specificity of DNA methylation perturbations following PB-treatment could be conferred by the nuclear receptor CAR, which upon PB-induction is recruited to the PBREM element in the *Cyp2b10* promoter [37]. CAR in turn could regulate the exclusion of DNA methyltransferases (DNMTs) and/or recruit DNA demethylases and different histone modifying enzymes to the *Cyp2b10* promoter (for a review on the role of histone modifying enzymes in nuclear receptor mediated gene regulation see Gronemeyer et al. [48]). The extent of chromatin remodeling observed through ChIP analyses points to the recruitment of co-activator complexes (histone acetyl transferase, trithorax-group complexes which mediate H3K4 di/tri-methylation) and exclusion of co-repressor complexes (including the Polycomb Repressive Complexes which mediate H3K27 tri-methylation) [49], [50] associated with *Cyp2b10* activation. Several lines of evidence point to functional antagonism between H3K4 methylation and DNA methylation [51], [52]. This antagonism, through the exclusion of DNMTs could explain *Cyp2b10* promoter DNA demethylation via a passive, DNA-replication dependent dilution of DNA methylation. We cannot exclude however that active promoter DNA demethylation takes place, as recently reported at the P450 gene *CYP27B1* [53].

The extent of observed DNA methylation perturbations at CpG islands in promoters and intergenic regions in this study is consistent with previously reported limited promoter-based DNA methylation remodeling in cancer cells [38]. Most cancer studies have assumed that functionally important DNA methylation will occur in CpG islands of promoters. Recent studies now suggest that proximal promoters may not be the most perturbed regions in cancer [54]. The development of new technologies such as whole-genome and reduced representation bisulfite sequencing further support a major role for non-promoter-based methylation (e.g. enhancer, intragenic) in regulating cell context-specific alternative promoters in gene bodies [55], [56], [57], [58]. Changes in liver DNA methylation have been previously reported following 4 weeks of PB treatment using a methodology based on methylation-sensitive enzyme digestion and arbitrary primed PCR reactions/capillary electrophoresis [34]. This method identified 86 regions of altered methylation (RAMs) upon 4 weeks of PB treatment. Our results now suggest that the majority of those DNA methylation perturbations take place outside of proximal promoter regions.

The profiling we report here allows for investigation of the epigenetic status of single genes and pathways that may be perturbed at different stages of xenobiotic exposure. This study contributes to understanding the scale and nature of drug-induced epigenetic changes in an *in vivo* setup relevant for drug safety assessment. Before epigenetic profiling can be included as an integral part of safety evaluation several practical and pragmatic issues need to be addressed [21], [25], [59]. Alternative DNA methylation profiling technologies (MeDIP-seq, whole genome bisulfite sequencing), further profiling of different chromatin marks and increased throughput through deep sequencing and high-density arrays as well as the analysis of a larger panel of (non)genotoxic carcinogens and non-carcinogen xenobiotics (http://www.imi-marcar.eu/) will provide deeper understanding of carcinogenic mechanisms and may lead to the identification of powerful predictive biomarkers.

## Materials and Methods

### Ethics Statement

This study was performed in conformity with the Swiss Animal Welfare Law and specifically under the Animal License No. 5041 by ‘Kantonales Veterinäramt Baselland’ (Cantonal Veterinary Office, Basel Land).

### Animal treatment and sample preparation

29–32 days old male B6C3F1/Crl (C57BL/6 ♂ x C3H/He ♀) mice were obtained from Charles River Laboratories (Germany). Animals were allowed to acclimatise for 5 days prior to being randomly divided into two treatment groups (n = 10) and phenobarbital (Sigma 04710, 0.05% (w/v) in drinking water) was administered to one group through *ad libitum* access to drinking water for 28 days. Mice were checked daily for activity and behavior and sacrificed on the last day of dosing (day 28). Blood was withdrawn for PK analysis and target (liver) and non-target (kidney) tissues removed, split into several sections, frozen in liquid nitrogen and stored at −80°C for subsequent analyses.

### RNA isolation

Frozen liver and kidney samples were homogenized in TRIzol reagent (Invitrogen) and subsequently purified on a silica-gel-based-membrane (RNeasy, Qiagen) according to the manufacturer's instructions. RNA quality was assessed by measuring the RIN (RNA Integrity Number) using an Agilent 2100 Bioanalyzer. RNA was stored at −80°C until GeneChip® experiment analysis.

### Affymetrix labeling and GeneChip processing

Processing of GeneChip® experiments was conducted as recommended by the manufacturer of the GeneChip® system (Affymetrix). For tissue samples, double stranded cDNA was synthesized with a starting amount of 0.1 µg total RNA. For RNA reverse transcription, the GeneChip® 3′ IVT Express Labelling Assay (lot ID 0904012, Affymetrix) was used in the presence of a T7-(dT)_24_ DNA oligonucleotide primer (Affymetrix). The cDNA was then transcribed *in vitro* in the presence of biotinylated ribonucleotides to form biotin-labelled amplified RNA (aRNA). The labelled aRNA was then purified and quantified by UV spectrophotometry at 260 nm and fragmented. 10 µg of fragmented, biotinylated aRNA were hybridized for approximately 16 hrs at 45°C to the GeneChip® Mouse430_2 arrays. The arrays were then washed and stained with the GeneChip® Hybridization Wash and Stain kit (Affymetrix). The washing and staining steps were performed with GeneChip® Fluidics Workstation 450 (Affymetrix). Arrays were then scanned using a solid-state laser scanner (GeneArray® Scanner 3000 combined with the GeneChip® autoloader, Affymetrix). The Affymetrix GeneChip® Operating Software (GCOS) was used to generate the primary and secondary raw data files.

### Processing of Affymetrix chips

Affymetrix chips were normalized with the MAS5 method (scaled to a trimmed mean of 150 per chip, Affymetrix, I. (2002) Statistical Algorithms Description Document http://www.affymetrix.com/support/technical/whitepapers.affx). For gene expression analysis, we excluded all probe sets with an average MAS5 signal of less than 50 in both groups (liver control and treated) to avoid large fold changes in genes that are not expressed under both of the experimental conditions. The cut-off of 50 is empirical, and we found that it performs better than the suggested present/absent calls of MAS5. This reduces the number of probe sets from 45,037 to 18,981 probes. We further excluded all probes with a small fold change (less extreme than +/− 1.5 fold) which results in 750 probe sets. We then filtered out probe sets with a p-value for the fold change (t-test) larger than 0.001 (expecting less than one false positive in 750 tests), resulting in 349 probes sets. This filtered list of probe set was used as input for ingenuity pathway analysis (Ingenuity Pathway Analysis, http://www.ingenuity.com/), and summarizes the 349 probe sets into 231 known genes.

### MeDIP assay

Genomic DNA from liver and kidney tissue samples was prepared by overnight Proteinase K (pK) treatment in lysis buffer (10 mM Tris-HCl pH 8.0, 50 mM EDTA pH 8.0, 100 mM NaCl, 0.5% SDS), phenol-chloroform extraction, ethanol precipitation and RNaseA digestion. Genomic DNA was sonicated (Bioruptor, Diagenode) to produce random fragments ranging in size from 300 to 1,000 bp and 6 µg of fragmented DNA was used for a standard MeDIP assay [38], [60]. DNA was denatured for 10 min at 95°C and immunoprecipitated for 2 hrs at 4°C with 10 µl of monoclonal antibody against 5-methylcytidine (Eurogentec) in a final volume of 500 µl IP buffer (10 mM sodium phosphate (pH 7.0), 140 mM NaCl, 0.05% Triton X-100). The mixture was incubated with 60 µl magnetic beads (Dynabeads M-280 Sheep anti-mouse IgG (Invitrogen) for 2 hrs at 4°C and washed three times with 700 µl of IP buffer. Beads were subsequently treated with pK for 3 hrs at 50°C and the methylated DNA recovered by phenol-chloroform extraction followed by ethanol precipitation. For qPCR, 12 ng sonicated genomic input DNA and 1/40 of a MeDIP reaction was used. For microarray analysis the genomic input DNA and MeDIP enriched DNA was amplified using WGA2: GenomePlex Complete Whole Genome kit (Sigma) and 3 µg of DNA was sent to Roche Nimblegen (Madison, USA) for Cy3 and Cy5 labeling and hybridization on mouse promoter tiling arrays.

### Processing of Nimblegen chips

The Nimblegen chips (385K CpG Island Plus Promoter Arrays) contain 373,683 probes of about 50 nucleotides length distributed over promoter regions of known genes, some intragenic regions and non-gene/non-promoter CpG islands. On average there are 16 probes tiled with a spacing of 100 nucleotides per regions, and we refer to these regions as probe sets. Chips were processed as described in Mohn et al. [3]. Briefly, after graphical QC, fold changes per chip and probe of IP DNA (enriched) over input DNA were calculated from the red (Cy5) and green (Cy3) channels as log_2_(IP/total), this is also known as an M-value. The raw M-values were then normalized to correct for saturation effects and dye bias using per array Loess normalization from the BioConductor package Limma [61]. Probes with a raw input signal (Cy3) of more than 15,000 are considered completely saturated and were excluded from further analysis. Per promoter the average signal was calculated within a window of −800 to +100 nucleotides of the transcription start (for alternative transcription starts the median was taken). The median M-value per chip was subtracted from each M-value, and all chips brought to the same scale (using BioConductor Limma normalizeBetweenArrays and scaling). These normalized average relative enrichments per promoter are the basis for all downstream analyses. For further analysis probe sets were filtered by a p-value (t-test) of ≤0.01 and a minimum absolute log_2_ fold change of 0.2.

Analysis of variance (ANOVA) was performed on a multi-variate model to evaluate the methylation perturbation by the different experimental factors and to evaluate the technical error introduced by the array platform. We did not include any signal fold change filtering as we did above. Our model includes organ and treatment (main effects), organ and treatment interaction and donor as random effect for each of the 23,428 probe sets. From each donor two samples were processed, one for liver and one for kidney and each of these samples hybridized on two arrays (technical replicates). The standard error between donors of the same group is on average twice as high as within donors. Since the technical variation (within donor) is low compared to the biological variation we took the mean per donor within each group (organ and treatment) for further fold change analyses (this reduces the degrees of freedom per group from 20 to 10 but observations are independent).

### Annotation of Nimblegen probes

Nimblegen probes and transcripts from ENSEMBL (version 46) and RefSeq (UCSC MM8) were mapped based on coordinates of the mouse genome assembly MM8. Of the 23,428 Nimblegen probe sets 17,967 are in the promoter regions of known genes (2,240 from RefSeq only, 1,610 from Ensembl only and 14,117 from both RefSeq and Ensembl). Affymetrix and Nimblegen probes were mapped by the gene symbols from NetAffx (http://www.affymetrix.com/analysis/index.affx) and our Nimblegen annotation database. A gene can have several Affymetrix probes. In this case we associate one methylation value with each expression value of the probeset for the mapped gene.

### TaqMan PCR

Total mouse liver/kidney RNA was reverse transcribed to cDNA using the High Capacity cDNA Archive Kit (Applied Biosystems, cat n°4322171) according to the manufacturer instructions. TaqMan based real time PCR method was performed with the TaqMan® Fast Universal PCR Master Mix (Applied Biosystems, cat n°4352042). The PCR cycling conditions wewre as follows: 1 cycle (95°C for 20 sec) and 40 cycles (95°C for 1 sec, 60°C for 20 sec). The following probe/primer sets were commercially purchased from Applied Biosystems: Mm01972453_s1, NM_009999.3 and 18S, Hs99999901_m1. The relative quantification of gene expression changes were performed using the comparative threshold (CT) method (ΔΔCT).

### Pyrosequencing

Genomic DNA was bisulfite treated using the EZ DNA Methylation™ Kit (ZYMO Research). 228 bp region around -900 bp at promoter containing 2 CpGs was amplified by PCR using biotinylated reverse primer. Biotinylated PCR products were purified and immobilized onto streptavidin-coated Sepharose beads (GE Healthcare). The pyrosequencing was done with Pyromark HD (Biotage) system according to manufacturer's instructions. Primers for PCR amplification and sequencing are listed in Table S9.

### Bisulfite DNA sequencing analysis

Genomic DNA (500 ng per column) was bisulfite treated using the EZ DNA Methylation™ Kit (ZYMO Research). Regions of interests were amplified by PCR and amplicons purified using the GenElute PCR Clean-Up Kit (Sigma). PCR amplicons were sub-cloned using the pGEM-T Vector System (Promega) and plasmids forming individual clones were amplified using the QIAprep Spin Miniprep Kit (QIAGEN) and examined by DNA sequencing (ABI). Sequences extracted from individual clones were then analyzed in BiQ Analyzer (http://biq-analyzer.bioinf.mpi-inf.mpg.de/) to reveal the status of CpG methylation. All steps were performed according to manufacturers's instructions. Primers for PCR amplification are listed in Table S9.

### Reverse Protein Array (RPA)

Liver and kidney samples for RPA analysis were prepared according to Zeptosens proprietary protocols. RPA analysis and data analysis of randomized samples was performed by Zeptosens (Zeptosens, Witterswil, CH).

### Native Chromatin Immunoprecipitation

N-ChIP protocol was based on a published protocol [43] with some modifications. ∼200 mg of frozen mouse liver was pulverized with Covaris Cryoprep (Covaris Inc, USA). Tissue was moved to Covaris TC16 tubes and 5 ml of ice cold Buffer I was added. Tissue was homogenized using Covaris E210 sonicator. Cell suspension was transferred to a 14 ml polypropylene tube and cells were spun down at 6000 g for 10 min, at 4°C. The pellet was re-suspended in 2 ml of ice-cold Buffer I. 2 ml of ice-cold Buffer II was added, mixed gently, and placed on ice for 10 min. 14 ml polypropylene tubes containing each 8 ml of ice-cold Buffer III were prepared. 2 ml of each cell suspension was laid on top of 8 ml sucrose cushion. Tubes were covered with a piece of Parafilm and centrifuged at 10,000 g, for 20 min at 4°C. Nuclei pellet was resuspended in 500 µl of digestion buffer. 0.125 U of MNase enzyme (#N3755, Sigma) was added to each tube and mixed gently. Digestion mixes were incubated at 28°C heatblock for 6 min shaking at 800 rpm. Digestion was stopped by adding EDTA to final concentration of 10 µM and tubes were left on ice for at least 5 min. Non-soluble fractions were removed by centrifuging at 10,000 rpm (+4°C) for 10 min and collecting the supernatant. The pellet was discarded. 10 µg of chromatin was used for each antibody for the immunoprecipitation. Antibodies used were IgG (Santa Cruz #sc-2027), H3K4me2 (Millipore #07-030), H3K27me3 (Millipore, #07-449) and H3K9ac (Millipore #07-352). The immunoprecipitation, washes and DNA purification was done with Magna ChIP™ A Chromatin Immunoprecipitation Kit (Millipore #17-610) following manufacturer's protocol. Quantification of input and immunoprecipitated DNA was obtained by qPCR by using SYBR Green detection on an ABI PRISM SDS 7900 HT machine (Applied Biosystems). Primers and conditions used are listed in Table S9.

### End Note

The complete data set is available from data files. These include data files for average liver and kidney treated and control methylation M-values (enrichment) in promoters including p-values and group differences as well as correlation of promoter methylation and gene expression. Also, the normalized and averaged per-promoter methylation signals are available per sample in addition to the raw files from Nimblegen chips. Affymetrix data will be submitted to GEO.

## Supporting Information

Figure S1 (**A**) Hierarchical clustering for the 349 Affymetrix probe sets that show differential expression upon PB treatment in the liver (used for the Ingenuity pathway analysis in Figure S1B). The color code indicates down-regulation (green), up-regulation (red) or no change (grey). (**B**) Ingenuity Pathway Analysis (www.ingenuity.com) of genes with differential expression upon 4-week PB-treatment. The table shows selected over-represented functional IPA categories. The full list of filtered differentially expressed genes is provided in Table S2 and the genes belonging to the different IPA categories are indicated in Table S3.
(EPS)

Figure S2 (**A**) Volcano plot analysis illustrating the extent of DNA methylation perturbation in liver and kidney. We used this plot to choose our empirical differential methylation and significance cut-off as described in materials and methods (Processing of Nimblegen chips). Only probe sets indicated in red (in the upper left and right rectangles of the plot) are considered differentially methylated (28 in liver and 286 in kidney) (**B**) Methylation comparison between kidneys of control and PB-treated mice (average log_2_ (IP/total) for replicates). The colors indicate the CpG class for those probe sets for which the log_2_ ratio of PB-treated vs. Control methylation signal (difference in M-value) is significant (p≤0.01 and absolute log_2_ fold change of ≥0.2, 286 probe sets), and non-differentially methylated regions are indicated in grey. The black dot highlights *Cyp2b10* (not part of the 286 differentially methylated probe sets). (**C**) Methylation comparison between kidney control and liver control mice (average log_2_ (IP/total) for replicates). The colors indicate the CpG class for those probe sets with p≤0.01 and absolute log_2_ fold change of ≥0.2 (2,811 probe sets), and non-differentially methylated regions are indicated in grey. The black and orange dot highlights *Cyp2b10* (not part of the 2,811) and Cyp2c44 (part of the 2,811) respectively.
(EPS)

Figure S3 Promoter *Cyp2b10* demethylation is associated with transcriptional activation. (**A**) For the three Affymetrix probe sets of the *Cyp2b10* gene, the MAS5 expression signals are plotted against the M-values (log_2_ (IP/total)) for methylation. Only one methylation signal (the average over the *Cyp2b10* promoter) is used for all three expression measurements. (**B**) Per probe methylation M-value distribution (normalized) for liver and kidney, treated and control at their genomic locations in proximity of *Cyp2b10*. Each box represents the log_2_(IP/total) methylation signal distribution of a single probe for replicate chips, the median splits the box in half, dotted lines indicate the upper and lower quartiles respectively, outliers are not shown. The start of *Cyp2b10* (blue) as well as the region in which probes are averaged for the calculation of the promoter signal (yellow, see Processing of Nimblegen chips in the methods section) is indicated.
(EPS)

Figure S4 Relative average levels of 16 post-translational histone modifications in control and phenobarbital-treated B6C3F1 mice liver samples using reverse protein array. Average levels of 8 treated and 8 control animals are shown. An asterisk indicates low levels of the mark (<0.05 AU). Raw data are available in Table S7.
(EPS)

Table S1 Unfiltered liver Affymetrix data. The Mouse430_2 chip contains 45,037 probes. Only the probe sets with a minimum average MAS5 signal of 50 in either control or treated were included (18981 probe sets remaining).
(XLSX)

Table S2 Filtered liver Affymetrix data. The Mouse430_2 chip contains 45,037 probes. Only the probe sets with a minimum average MAS5 signal of 50 in either control or treated were included (18,981 probe sets remaining). The probe sets were further filtered to select regions with a 1.5 fold change (750 probe sets remaining) and a p-value≤0.001 (349 probe sets remaining). IPA identified 231 individual genes.
(XLSX)

Table S3 Ingenuity pathway analyses, listing the genes included in individual functional IPA categories (Figure S1B).
(XLSX)

Table S4 Filtered kidney Affymetrix data, as in Table S2.
(XLSX)

Table S5 Genomic coordinates of each of the 23,428 probe sets in the mouse MM8 genome, mean signal (log_2_(IP/total) over replicates, fold change between control and treated means and the p-value for the fold change (t-test) for liver and kidney respectively, also given is the gene symbol or Ensembl gene Id (for those entries without an EntrezGene entry) and the CpG class The table can be filtered by p-value (≤0.01) and fold change (≥0.2 or ≤−0.2) to extract our 28 probe sets for liver.
(XLSX)

Table S6 Bioinformatics integration of all Affymetrix and MeDIP-array data.This tables lists all genes that are annotated with at least one Affymetrix probe set and one Nimblegen probe set and includes the mean MAS5 expression signal and methylation signals over replicates in each group, the fold change and p-value.
(XLS)

Table S7 Reverse protein array results measuring relative levels of 16 post-translational histone modifications in the liver and kidney from 8 control and 8 Phenobarbital-treated B6C3F1 mice. Average expression levels are represented as arbitrary units (with standard deviation) and can only be compared between organs/treatment, not between different protein endpoints.
(DOCX)

Table S8 Reverse protein array results measuring relative levels of 31 (phospho)-proteins in the liver and kidney from 8 control and 8 Phenobarbital-treated B6C3F1 mice. Average expression levels are represented as arbitrary units (with standard deviation) and can only be compared between organs/treatment, not between different protein endpoints.
(DOCX)

Table S9 List of primers used in the expression and epigenetic assays.
(DOCX)

## References

[1] Bernstein BE, Meissner A, Lander ES (2007). The mammalian epigenome.. Cell.

[2] Reik W (2007). Stability and flexibility of epigenetic gene regulation in mammalian development.. Nature.

[3] Mohn F, Weber M, Rebhan M, Roloff TC, Richter J (2008). Lineage-specific polycomb targets and de novo DNA methylation define restriction and potential of neuronal progenitors.. Mol Cell.

[4] Strahl BD, Allis CD (2000). The language of covalent histone modifications.. Nature.

[5] Jenuwein T, Allis CD (2001). Translating the histone code.. Science.

[6] Cedar H, Bergman Y (2009). Linking DNA methylation and histone modification: patterns and paradigms.. Nat Rev Genet.

[7] Jones PL, Wolffe AP (1999). Relationships between chromatin organization and DNA methylation in determining gene expression.. Semin Cancer Biol.

[8] Khorasanizadeh S (2004). The nucleosome: from genomic organization to genomic regulation.. Cell.

[9] Wolffe AP (1994). Inheritance of chromatin states.. Dev Genet.

[10] Bird A (2007). Perceptions of epigenetics.. Nature.

[11] Handel AE, Ebers GC, Ramagopalan SV (2010). Epigenetics: molecular mechanisms and implications for disease.. Trends Mol Med.

[12] Feinberg AP, Vogelstein B (1983). Hypomethylation distinguishes genes of some human cancers from their normal counterparts.. Nature.

[13] Goelz SE, Vogelstein B, Hamilton SR, Feinberg AP (1985). Hypomethylation of DNA from benign and malignant human colon neoplasms.. Science.

[14] Ehrlich M (2009). DNA hypomethylation in cancer cells.. Epigenomics.

[15] Fraga MF, Ballestar E, Villar-Garea A, Boix-Chornet M, Espada J (2005). Loss of acetylation at Lys16 and trimethylation at Lys20 of histone H4 is a common hallmark of human cancer.. Nat Genet.

[16] Chi P, Allis CD, Wang GG (2010). Covalent histone modifications–miswritten, misinterpreted and mis-erased in human cancers.. Nat Rev Cancer.

[17] Baylin SB, Ohm JE (2006). Epigenetic gene silencing in cancer - a mechanism for early oncogenic pathway addiction?. Nat Rev Cancer.

[18] Feinberg AP, Ohlsson R, Henikoff S (2006). The epigenetic progenitor origin of human cancer.. Nat Rev Genet.

[19] Jones PA, Baylin SB (2007). The epigenomics of cancer.. Cell.

[20] Bombail V, Moggs JG, Orphanides G (2004). Perturbation of epigenetic status by toxicants.. Toxicol Lett.

[21] Lebaron MJ, Rasoulpour RJ, Klapacz J, Ellis-Hutchings RG, Hollnagel HM (2010). Epigenetics and chemical safety assessment.. Mutat Res.

[22] Phillips JM, Yamamoto Y, Negishi M, Maronpot RR, Goodman JI (2007). Orphan nuclear receptor constitutive active/androstane receptor-mediated alterations in DNA methylation during phenobarbital promotion of liver tumorigenesis.. Toxicol Sci.

[23] Watson RE, Goodman JI (2002). Epigenetics and DNA methylation come of age in toxicology.. Toxicol Sci.

[24] Esteller M (2007). Cancer epigenomics: DNA methylomes and histone-modification maps.. Nat Rev Genet.

[25] Csoka AB, Szyf M (2009). Epigenetic side-effects of common pharmaceuticals: a potential new field in medicine and pharmacology.. Med Hypotheses.

[26] Peraino C, Fry RJ, Staffeldt E (1971). Reduction and enhancement by phenobarbital of hepatocarcinogenesis induced in the rat by 2-acetylaminofluorene.. Cancer Res.

[27] Peraino C, Fry RJ, Staffeldt E (1973). Brief communication: Enhancement of spontaneous hepatic tumorigenesis in C3H mice by dietary phenobarbital.. J Natl Cancer Inst.

[28] Lee GH (2000). Paradoxical effects of phenobarbital on mouse hepatocarcinogenesis.. Toxicol Pathol.

[29] Becker FF (1982). Morphological classification of mouse liver tumors based on biological characteristics.. Cancer Res.

[30] Kawamoto T, Sueyoshi T, Zelko I, Moore R, Washburn K (1999). Phenobarbital-responsive nuclear translocation of the receptor CAR in induction of the CYP2B gene.. Mol Cell Biol.

[31] Yamamoto Y, Kawamoto T, Negishi M (2003). The role of the nuclear receptor CAR as a coordinate regulator of hepatic gene expression in defense against chemical toxicity.. Arch Biochem Biophys.

[32] Phillips JM, Burgoon LD, Goodman JI (2009a). The constitutive active/androstane receptor facilitates unique phenobarbital-induced expression changes of genes involved in key pathways in precancerous liver and liver tumors.. Toxicol Sci.

[33] Ross J, Plummer SM, Rode A, Scheer N, Bower CC (2010). Human Constitutive Androstane Receptor (CAR) and Pregnane X Receptor (PXR) Support the Hypertrophic but not the Hyperplastic Response to the Murine Nongenotoxic Hepatocarcinogens Phenobarbital and Chlordane In Vivo.. Toxicol Sci.

[34] Bachman AN, Phillips JM, Goodman JI (2006). Phenobarbital induces progressive patterns of GC-rich and gene-specific altered DNA methylation in the liver of tumor-prone B6C3F1 mice.. Toxicol Sci.

[35] Phillips JM, Burgoon LD, Goodman JI (2009b). Phenobarbital elicits unique, early changes in the expression of hepatic genes that affect critical pathways in tumor-prone B6C3F1 mice.. Toxicol Sci.

[36] Phillips JM, Goodman JI (2009). Multiple genes exhibit phenobarbital-induced constitutive active/androstane receptor-mediated DNA methylation changes during liver tumorigenesis and in liver tumors.. Toxicol Sci.

[37] Honkakoski P, Zelko I, Sueyoshi T, Negishi M (1998). The nuclear orphan receptor CAR-retinoid X receptor heterodimer activates the phenobarbital-responsive enhancer module of the CYP2B gene.. Mol Cell Biol.

[38] Weber M, Davies JJ, Wittig D, Oakeley EJ, Haase M (2005). Chromosome-wide and promoter-specific analyses identify sites of differential DNA methylation in normal and transformed human cells.. Nat Genet.

[39] Honkakoski P, Kojo A, Lang MA (1992). Regulation of the mouse liver cytochrome P450 2B subfamily by sex hormones and phenobarbital.. Biochem J.

[40] Honkakoski P, Negishi M (1997). Characterization of a phenobarbital-responsive enhancer module in mouse P450 Cyp2b10 gene.. J Biol Chem.

[41] Moncman CL, Wang K (1995). Nebulette: a 107 kD nebulin-like protein in cardiac muscle.. Cell Motil Cytoskeleton.

[42] Leek JT, Scharpf RB, Bravo HC, Simcha D, Langmead B (2010). Tackling the widespread and critical impact of batch effects in high-throughput data.. Nat Rev Genet.

[43] Umlauf D, Goto Y, Feil R (2004). Site-specific analysis of histone methylation and acetylation.. Methods Mol Biol.

[44] Klug M, Heinz S, Gebhard C, Schwarzfischer L, Krause SW (2010). Active DNA demethylation in human postmitotic cells correlates with activating histone modifications, but not transcription levels.. Genome Biol.

[45] Uchida E, Hirono I (1981). Effect of phenobarbital on the development of neoplastic lesions in the liver of cycasin-treated rats.. J Cancer Res Clin Oncol.

[46] Hirose M, Shirai T, Tsuda H, Fukushima S, Ogiso T (1981). Effect of phenobarbital, polychlorinated biphenyl and sodium saccharin on hepatic and renal carcinogenesis in unilaterally nephrectomized rats given N-ethyl-N-hydroxyethylnitrosamine orally.. Carcinogenesis.

[47] Gachon F, Olela FF, Schaad O, Descombes P, Schibler U (2006). The circadian PAR-domain basic leucine zipper transcription factors DBP, TEF, and HLF modulate basal and inducible xenobiotic detoxification.. Cell Metab.

[48] Gronemeyer H, Gustafsson JA, Laudet V (2004). Principles for modulation of the nuclear receptor superfamily.. Nat Rev Drug Discov.

[49] Schuettengruber B, Chourrout D, Vervoort M, Leblanc B, Cavalli G (2007). Genome regulation by polycomb and trithorax proteins.. Cell.

[50] Schwartz YB, Pirrotta V (2007). Polycomb silencing mechanisms and the management of genomic programmes.. Nat Rev Genet.

[51] Weber M, Hellmann I, Stadler MB, Ramos L, Paabo S (2007). Distribution, silencing potential and evolutionary impact of promoter DNA methylation in the human genome.. Nat Genet.

[52] Ooi SK, Qiu C, Bernstein E, Li K, Jia D (2007). DNMT3L connects unmethylated lysine 4 of histone H3 to de novo methylation of DNA.. Nature.

[53] Kim MS, Kondo T, Takada I, Youn MY, Yamamoto Y (2009). DNA demethylation in hormone-induced transcriptional derepression.. Nature.

[54] Irizarry RA, Ladd-Acosta C, Wen B, Wu Z, Montano C (2009). The human colon cancer methylome shows similar hypo- and hypermethylation at conserved tissue-specific CpG island shores.. Nat Genet.

[55] Schmidl C, Klug M, Boeld TJ, Andreesen R, Hoffmann P (2009). Lineage-specific DNA methylation in T cells correlates with histone methylation and enhancer activity.. Genome Res.

[56] Lister R, Pelizzola M, Dowen RH, Hawkins RD, Hon G (2009). Human DNA methylomes at base resolution show widespread epigenomic differences.. Nature.

[57] Maunakea AK, Nagarajan RP, Bilenky M, Ballinger TJ, D'Souza C (2010). Conserved role of intragenic DNA methylation in regulating alternative promoters.. Nature.

[58] Wu H, Coskun V, Tao J, Xie W, Ge W (2010). Dnmt3a-dependent nonpromoter DNA methylation facilitates transcription of neurogenic genes.. Science.

[59] Goodman JI, Augustine KA, Cunnningham ML, Dixon D, Dragan YP (2010). What do we need to know prior to thinking about incorporating an epigenetic evaluation into safety assessments?. Toxicol Sci.

[60] Mohn F, Weber M, Schubeler D, Roloff TC (2009). Methylated DNA immunoprecipitation (MeDIP).. Methods Mol Biol.

[61] Smyth GK (2004). Linear models and empirical bayes methods for assessing differential expression in microarray experiments.. Stat Appl Genet Mol Biol.

