# Supplementary material for: Phenobarbital Mediates an Epigenetic Switch at the Constitutive Androstane Receptor (CAR) Target Gene Cyp2b10 in the Liver of B6C3F1 Mice
Source: PLoS One. 2011 Mar 24;6(3):e18216. doi: 10.1371/journal.pone.0018216 (PMC3063791; doi:10.1371/journal.pone.0018216)
Supplement: Table S7 — Reverse protein array results measuring relative levels of 16 post-translational histone modifications in the liver and kidney from 8 control and 8 Phenobarbital-treated B6C3F1 mice. Average expression levels are represented as arbitrary units (with standard deviation) and can only be compared between organs/treatment, not between different protein endpoints. (DOCX) [file pone.0018216.s011.docx]

Table S7: Relative level of 16 histone modifications in the liver and kidney from 8 control and 8 Phenobarbital-treated B6C3F1 mice. Average expression levels are given as arbitrary units (with standard deviation) that can only be compared between organs/treatment, not between different protein endpoints.

| endpoint | Liver | | Kidney | |
| --- | --- | --- | --- | --- |
|  | control | Phenobarbital | control | Phenobarbital |
| H2AK9ac | 0.56 (0.10) | 0.53 (0.08) | 0.60 (0.07) | 0.67 (0.08) |
| H2BK15ac | 0.17 (0.03) | 0.19 (0.04) | 0.31 (0.05) | 0.33 (0.03) |
| H2BK20ac | 0.86 (0.12) | 0.72 (0.10) | 1.04 (0.13) | 1.06 (0.09) |
| H3K9ac | 0.04 (0.01) | 0.02 (0.01) | 0.03 (0.01) | 0.04 (0.01) |
| H3K23ac | 0.06 (0.01) | 0.05 (0.01) | 0.13 (0.03) | 0.12 (0.03) |
| H3K27ac | 0.02 (0.00) | 0.02 (0.00) | 0.03 (0.00) | 0.03 (0.00) |
| H3K9ac,K14ac | 1.09 (0.15) | 1.00 (0.24) | 1.99 (0.38) | 1.96 (0.34) |
| H3K4me | 0.30 (0.07) | 0.25 (0.05) | 0.47 (0.09) | 0.53 (0.06) |
| H3R17me2 | 0.21 (0.03) | 0.17 (0.02) | 0.12 (0.03) | 0.13 (0.03) |
| H3K36me2 | 0.17 (0.09) | 0.14 (0.10) | 0.18 (0.08) | 0.36 (0.11) |
| H3K79me2 | 0.52 (0.15) | 0.49 (0.15) | 0.78 (0.19) | 0.79 (0.12) |
| H3K4me3 | 0.04 (0.01) | 0.03 (0.01) | 0.06 (0.02) | 0.06 (0.01) |
| H3K9me3 | 0.19 (0.01) | 0.19 (0.01) | 0.22 (0.03) | 0.23 (0.03) |
| H4K12ac | 0.04 (0.01) | 0.03 (0.01) | 0.06 (0.01) | 0.07 (0.01) |
| H4K20me2 | 0.02 (0.00) | 0.02 (0.00) | 0.03 (0.01) | 0.03 (0.00) |
| H4K20me3 | 3.24 (0.37) | 2.86 (0.27) | 3.33 (0.29) | 3.46 (0.36) |
