# Supplementary material for: Phenobarbital Mediates an Epigenetic Switch at the Constitutive Androstane Receptor (CAR) Target Gene Cyp2b10 in the Liver of B6C3F1 Mice
Source: PLoS One. 2011 Mar 24;6(3):e18216. doi: 10.1371/journal.pone.0018216 (PMC3063791; doi:10.1371/journal.pone.0018216)
Supplement: Table S8 — Reverse protein array results measuring relative levels of 31 (phospho)-proteins in the liver and kidney from 8 control and 8 Phenobarbital-treated B6C3F1 mice. Average expression levels are represented as arbitrary units (with standard deviation) and can only be compared between organs/treatment, not between different protein endpoints. (DOCX) [file pone.0018216.s012.docx]

Table S8: Relative level of 31 (phospho)-proteins in the liver and kidney from 8 control and 8 Phenobarbital-treated B6C3F1 mice. Average expression levels are given as arbitrary units (with standard deviation) that can only be compared between organs/treatment, not between different protein endpoints.

| endpoint | Liver | | Kidney | |
| --- | --- | --- | --- | --- |
|  | control | Phenobarbital | control | Phenobarbital |
| AMPKα | 1.19 (0.17) | 1.29 (0.17) | 0.64 (0.06) | 0.65 (0.06) |
| AMPKα(p-Thr172) | 0.15 (0.02) | 0.15 (0.03) | 0.16 (0.01) | 0.18 (0.01) |
| AKT | 0.27 (0.04) | 0.25 (0.03) | 0.43 (0.07) | 0.42 (0.07) |
| AKT(p-Ser473) | 0.09 (0.02) | 0.08 (0.01) | 0.09 (0.01) | 0.09 (0.01) |
| AKT(p-Thr308) | 1.25 (0.20) | 1.17 (0.16) | 1.13 (0.08) | 1.12 (0.15) |
| ATM/ATR substrate (p-Ser/Thr) | 0.15 (0.03) | 0.14 (0.02) | 0.17 (0.03) | 0.20 (0.03) |
| BAD(p-Ser136) | 0.05 (0.01) | 0.06 (0.01) | 0.04 (0.01) | 0.04 (0.01) |
| c-JUN(p-Ser74) | 0.04 (0.01) | 0.03 (0.01) | 0.06 (0.01) | 0.05 (0.01) |
| ChK2(p-Thr68) | 0.02 (0.00) | 0.02 (0.01) | 0.01 (0.01) | 0.01 (0.01) |
| 4E-BP1(p-Ser65) | 0.06 (0.01) | 0.07 (0.01) | 0.04 (0.01) | 0.04 (0.01) |
| CREB | 0.19 (0.03) | 0.18 (0.02) | 0.19 (0.01) | 0.19 (0.02) |
| CREB(p-Ser133) | 0.05 (0.01) | 0.04 (0.01) | 0.07 (0.01) | 0.07 (0.01) |
| FKHR(p-Ser256) | 1.52 (0.11) | 1.32 (0.07) | 0.48 (0.04) | 0.44 (0.03) |
| GSK3β(p-Ser9) | 0.07 (0.01) | 0.06 (0.01) | 0.04 (0.01) | 0.05 (0.01) |
| mTOR | 0.54 (0.03) | 0.60 (0.07) | 0.46 (0.05) | 0.44 (0.03) |
| mTOR(p-Ser2448) | 0.02 (0.00) | 0.01 (0.01) | 0.01 (0.01) | 0.01 (0.01) |
| p53 | 0.12 (0.01) | 0.12 (0.01) | 0.15 (0.01) | 0.15 (0.02) |
| p53(p-Ser15) | 0.10 (0.01) | 0.11 (0.01) | 0.13 (0.01) | 0.13 (0.01) |
| STAT1 | 0.86 (0.08) | 0.75 (0.09) | 0.44 (0.06) | 0.43 (0.03) |
| STAT1(p-Ser73) | 0.79 (0.11) | 0.63 (0.11) | 0.80 (0.09) | 0.81 (0.10) |
| STAT3 | 1.93 (0.28) | 1.65 (0.25) | 0.05 (0.01) | 0.05 (0.00) |
| STAT3(p-Tyr75) | 0.15 (0.03) | 0.11 (0.02) | 0.09 (0.01) | 0.09 (0.01) |
| SAPK/JNK(p-Thr183,Tyr185) | 0.75 (0.12) | 0.66 (0.14) | 0.76 (0.16) | 0.77 (0.11) |
| MEK(p-Ser217, 221) | 1.28 (0.30) | 1.10 (0.23) | 0.88 (0.08) | 0.93 (0.13) |
| IRS1 | 0.18 (0.02) | 0.18 (0.02) | 0.20 (0.02) | 0.21 (0.03) |
| Tyk2 | 0.28 (0.02) | 0.30 (0.04) | 0.28 (0.03) | 0.28 (0.02) |
| Tyk2(p-Tyr1054,1055) | 0.15 (0.01) | 0.15 (0.01) | 0.18 (0.02) | 0.19 (0.02) |
| p38 MAPK(p-Thr180,Tyr182) | 0.13 (0.04) | 0.17 (0.07) | 0.16 (0.01) | 0.14 (0.01) |
| P44/42 MAPK (p-Thr202,Tyr204) | 0.05 (0.03) | 0.03 (0.02) | 0.03 (0.01) | 0.04 (0.01) |
| p70 S6 kinase(p-Thr389) | 0.26 (0.03) | 0.26 (0.05) | 0.12 (0.02) | 0.13 (0.02) |
| p70 S6 kinase(p-Thr421,Ser424) | 0.28 (0.05) | 0.21 (0.07) | 0.12 (0.01) | 0.12 (0.02) |
