# Supplementary material for: Phenobarbital Mediates an Epigenetic Switch at the Constitutive Androstane Receptor (CAR) Target Gene Cyp2b10 in the Liver of B6C3F1 Mice
Source: PLoS One. 2011 Mar 24;6(3):e18216. doi: 10.1371/journal.pone.0018216 (PMC3063791; doi:10.1371/journal.pone.0018216)
Supplement: Table S9 — List of primers used in the expression and epigenetic assays. (DOCX) [file pone.0018216.s013.docx]

**Table S9.** List of primers used in the expression and epigenetic assays different assays

| **Application** | **Gene** | **Sequence 5’-3’** | **Tm (°C)** | **Position** |
| --- | --- | --- | --- | --- |
| **MeDIP, ChIP** | **Gapdh** | TCCCTAGACCCGTACAGTGC | 60 | Promoter |
|  |  | CTCTGCTCCTCCCTGTTCC |  |  |
|  | **Beta-actin** | AGCCAACTTTACGCCTAGCGT | 60 | Promoter |
|  |  | TCTCAAGATGGACCTAATACGGC |  |  |
|  | **Hoxa9** | GGAGGGAGGGGAGTAACAAA | 60 | Promoter |
|  |  | TCACCTCGCCTAGTTTCTGG |  |  |
|  | **Oct4** | TGGGCTGAAATACTGGGTTC | 60 | Promoter |
|  |  | GTCCTTACAGCCCACTCAG |  |  |
|  | **Cyp2b10_promoter_5’** | CATGGAGGGATGCTGCTTAC | 60 | Promoter |
|  |  | TGTTGGAGGGCCTAAGTCAC |  |  |
|  | **Cyp2b10_promoter_3’** | GGCCAGCATAAAAGATCCTG | 60 | Promoter |
|  |  | CAAGAAGCCCACAAGGAGAG |  |  |
|  | **Cyp2b10_Intron1_5’** | GGAACCTCTTGCAGATGGAC | 60 | Exon-Intron1 |
|  |  | AGGGGGAAACCCACACTAAG |  |  |
|  | **Cyp2b10_Intron1_3’** | TCCTTCCACGGTGTAGGTTC | 60 | Intron1 |
|  |  | CCACACAACTCCAACGTAGC |  |  |
|  | **H19 ICR** | GCATGGTCCTCAAATTCTGCA | 60 |  |
|  |  | GCATCTGAACGCCCCAATTA |  |  |
|  | **IAP** | CTCCATGTGCTCTGCCTTCC | 60 |  |
|  |  | CCCCGTCCCTTTTTTAGGAGA |  |  |
|  | **Intergenic3** | ATGCCCCTCAGCTATCACAC | 60 |  |
|  |  | GGACAGACATCTGCCAAGGT |  |  |
|  | **CSa** | TGGTTGGCATTTTATCCCTAGAAC | 60 |  |
|  |  | GCAACATGGCAACTGGAAACA |  |  |
|  | **HPRT** | CCAAGACGACCGCATGAGAG | 60 | Promoter |
|  |  | CAACGGAGTGATTGCGCATT |  |  |
|  | **Scarb1** | TTGCACACCCTTCGTACAG | 60 | Promoter |
|  |  | TAAGCCCCTCGAACACATTC |  |  |
|  | **Klotho-beta** | TGTAACCCCAGGCTGACTTC | 60 | Promoter |
|  |  | ATTTCCAAGACCCCCAAATC |  |  |
|  | **Sfi1** | TTCCATGGTACAAACGCAAC | 60 | Promoter |
|  |  | AGATCCACGTTCGATTCAGG |  |  |
| **Bisulfite sequencing** | **Cyp2b10** | GTAGATGGATAGAGGAGGTTTTTTTAAG | 68.7 | Promoter |
|  |  | ACAAACTCCAAAAAATAAAATTCTACAC |  |  |
|  | **H19 ICR** | GTTGTGTAGATTTGGTTATAGTTA | 53 |  |
|  |  | TTCTCCTAATCTCTAATCTCAA |  |  |
|  | **Klotho β** | GGAATGAATGGATTTTTTTTAGTTTT | 68 | Promoter |
|  |  | AAATCCTCTTCCATCTATCTTCCA |  |  |
| **Pyro-sequencing** | **Cyp2b10** | **PCR_fw:** TTGTTTATTTATTATGGTTTGTTT AGTTTGGT  **PCR_rev:** ATGTGTTATGTTGATATAAAAG TTAGTT(biotinylated) | 60 | Promoter |
|  |  | **Seq:** TTAGGATTATTAGTTTAGGGATGAA |  |  |
| **RT-qPCR analysis** | Taqman(R) Gene Expression  Assays : **Cyp2b10** | Assay ID : Mm01972453_s1 |  | NCBI  NM_009999.3,AF128849.1,AF129405.1,BC060973.1,AK028103.1 |
|  |  |  |  |  |
